# Overview of medical errors and adverse events

**DOI:** 10.1186/2110-5820-2-2

**Published:** 2012-02-16

**Authors:** Maité Garrouste-Orgeas, François Philippart, Cédric Bruel, Adeline Max, Nicolas Lau, B Misset

**Affiliations:** 1 Réanimation médico-chirurgicale, Groupe Hospitalier Paris Saint Joseph, Paris, France; 2 Université Joseph Fourier, Unité INSERM, Epidémiologie des cancers et des maladies sévères, Institut Albert Bonniot, La Tronche, France; 3 Medicine Faculty, Université Paris Descartes, Paris, France; 4 Infection and Epidemiology department Pasteur Institut, Paris, France

## Abstract

Safety is a global concept that encompasses efficiency, security of care, reactivity of caregivers, and satisfaction of patients and relatives. Patient safety has emerged as a major target for healthcare improvement. Quality assurance is a complex task, and patients in the intensive care unit (ICU) are more likely than other hospitalized patients to experience medical errors, due to the complexity of their conditions, need for urgent interventions, and considerable workload fluctuation. Medication errors are the most common medical errors and can induce adverse events. Two approaches are available for evaluating and improving quality-of-care: the room-for-improvement model, in which problems are identified, plans are made to resolve them, and the results of the plans are measured; and the monitoring model, in which quality indicators are defined as relevant to potential problems and then monitored periodically. Indicators that reflect structures, processes, or outcomes have been developed by medical societies. Surveillance of these indicators is organized at the hospital or national level. Using a combination of methods improves the results. Errors are caused by combinations of human factors and system factors, and information must be obtained on how people make errors in the ICU environment. Preventive strategies are more likely to be effective if they rely on a system-based approach, in which organizational flaws are remedied, rather than a human-based approach of encouraging people not to make errors. The development of a safety culture in the ICU is crucial to effective prevention and should occur before the evaluation of safety programs, which are more likely to be effective when they involve bundles of measures.

## Introduction

During the past decade, healthcare quality and patient safety have emerged as major targets for improvement. Widely publicized reports from the United States, such as *Crossing the Quality Chasm *[1] and *To Err is Human *[2], showed that medical errors were common and adversely affected patient outcomes. These publications made the general public acutely aware of the inadequacies in the health care available to them. They also prompted healthcare providers, governments, and medical societies throughout the world to develop tools for measuring healthcare quality in all the fields of medicine. Institutions promoting error reporting were set up in Australia [3] and the United States [4] in 2000, in the United Kingdom in 2003 [5], and in France in 2006 [6].

The concept of quality has evolved from a process grounded in the physician-patient relationship to broader approaches involving the healthcare community, concept of efficiency, and ethical access to care. When discussing quality of care, it should be borne in mind that safety is a global concept encompassing efficiency, security of care, reactivity of caregivers, and satisfaction of patients and relatives. Starting in the 19^th ^century, several landmark events laid the foundation for the development of quality of care. During the Crimean war in the 1850s, Florence Nightingale studied mortality rates in military hospitals. In 1912, Ernest Codman developed a method to measure the outcomes of surgical interventions. In 1918, the American College of Surgery defined the minimum standard that hospitals needed to fulfil to obtain accreditation. In 1950, the medical audit method was developed by P. Lembcke in the United States and 1 year later the Joint Commission on Accreditation of Hospitals (JCAH) was created to accredit those hospitals that applied standard quality measures. In 1970, J. Williamson introduced a new method for assessing what is achievable but not achieved by the standard of care to what is actually done, via patient chart review and patient questionnaires. In 1992, Avedis Donabedian applied the industrial model of structure, process, and outcome measures to the healthcare process. Finally, H. Palmer defined the different dimensions of quality.

Quality assurance is a complex task, and patients in the intensive care unit (ICU) are more likely than other hospitalized patients to experience medical errors, due to the complexity of their conditions, need for urgent interventions, and considerable workload fluctuation [7-15]. Thus, the risk of medical errors associated with ICU admission deserves continuous attention. Safety must be defined and measurement tools devised. The indicators for routine monitoring must be clearly identified. The impact of medical errors and other adverse events on patients and relatives must be investigated. Prevention strategies must be developed and evaluated. The keys to developing a culture of patient safety in the ICU must be found. In this article, we review these points.

### Defining safety

In *To Err is Human *[2], safety is defined as freedom from accidental injury and error as failure of a planned action to be completed as intended (i.e., error of execution) or use of a wrong plan to achieve a goal (i.e., error of planning). Two types of execution errors exist: errors of commission (unintentionally doing the wrong thing) and errors of omission (unintentionally not doing the right thing). Errors can occur at any step of patient management, including diagnosis, treatment, and prevention.

An error may or may not cause an adverse event. Adverse events are injuries that result from a medical intervention and are responsible for harm to the patient (death, life-threatening illness, disability at the time of discharge, prolongation of the hospital stay, etc.) [2]. A near-miss is an adverse event that either resolves spontaneously or is neutralized by voluntary action before the consequences have time to develop. Adverse events may be due to medical errors, in which case they are preventable, or to factors that are not preventable.

### Measuring safety

There are two basic approaches to the evaluation and improvement of quality of care. In the room-for-improvement model, problems are identified, plans are then devised to correct the problems, and the effectiveness of the plans is assessed. This approach is known as the Plan-Do-Act Cycle (PDAC) of the Institute for Healthcare Improvement. The second way to measure safety is to use a monitoring system that detects problems and evaluates it periodically using quality indicators. These two approaches are complementary and often are used concomitantly. Thus, the monitoring model can be viewed as a way to seek opportunities for improvement by initiating a PDAC.

Safety measurement requires a self-assessment system for quantifying what we do and how we do it to help us to identify targets for improvement. A surveillance system needs multiple identification methods to detect medical errors and adverse events. These methods are implemented at the national or local level. National governments or agencies have developed reporting systems. At the hospital level, public and private agencies in North America have developed patient safety and improvement programs since 2005, as well as private databases to facilitate adverse-event reporting. In Europe, a safety program called The European Network for Patient Safety (EUNetPAS) was launched in 2008 to develop a culture of patient safety, provide a framework for education and training in patient safety, develop a core European curriculum on patient safety, implement reporting and learning systems, and implement methods to ensure medication safety. At the hospital level, different reporting systems are available to healthcare workers.

• The medical review. Reviews that do not target selected indicators are time-consuming and depend on the information available in the charts. Reviews can focus on selected indicators that can be assessed using the administrative data, discharge summaries, or mortality/morbidity review data. Medical reviews may be conducted manually or electronically using text words or text mining. Factors that may limit the use of the medical review method include absence of electronic medical records, paucity of resources for performing the reviews, variability in the terms used to label adverse events, and spelling mistakes. Failure to standardize the terminology may increase the difficulty of the search and the risk of false-positive results. Moreover, the analysis of documented adverse events requires considerable skill in interpreting the data. A meta-analysis comparing the rate of detection of pharmacists vs. nonpharmacists revealed a high level of adverse-event detection by pharmacists [16].

• Voluntary reporting is the method most often used to detect medical errors and adverse events. Limitations include underreporting due to time constraints, lack of adequate reporting systems, fear of litigation, a reluctance to report one's own errors, uncertainty of the clinical importance of the events, and the lack of changes after reporting. However, this reporting method is the most useful for inducing behavioral changes, demonstrating the benefits of adverse-event reporting, and allowing us to learn from our errors. The presence of a multidisciplinary safety team might facilitate voluntary reporting.

• Medical errors and adverse events also can be detected by direct observation at the bedside [17,18]. This method is useful for detecting errors by omission. For example, medication errors can occur at any stage of the medication process (prescription, delivery, dispensing, administration, and monitoring), Medication error rates varied in the studies according to the definitions used, the medication process being evaluated, and the method of reporting. A pharmacist at the bedside can collect errors by omissions not detected by voluntary reporting. The medication error rate varied from 7.45/1,000 patient-days with voluntary reporting to 560/1,000 patient-days with daily routine observation of prescriptions [10,12]. Similarly, the presence of a trained clinical research assistant who collected medical errors increased the rate from 2.2/1,000 to 597/1,000 patient-days in the IATROREF studies [14,19].

• The past several years have seen growing interest in learning from patients' experiences of care safety in all countries [20], with an older tradition in the United States and the United Kingdom via the CAHPS (Consumer Assessment of Healthcare Providers and Systems) and National Health Service (NHS), respectively. In 2007, the OECD (Organisation for Economic Co-operation and Development) established the patient's experience as a key priority. In the ICU, many patients are too ill to report on their own experience, but information can be obtained from families instead.

Combining these methods to ensure robust reporting of medical errors and adverse events is essential to obtain a global picture of care delivery in the ICU. The above-described surveillance systems require the use of valid indicators. Ideally, each indicator is expressed as a rate with a numerator (number of events, which can be defined easily and accurately) and a denominator (domain of care or population at risk). The surveillance system should include standardized data-collections forms, which should be used by trained staff. Data quality must be checked regularly (via audits and checks of missing data). Event rates may be difficult to determine when the definitions differ across institutions or medical societies or are not accepted by all leaders and when the at-risk population cannot be accurately determined.

According to Avedis Donabedian, three categories of indicators can be used: structure indicators (what we want vs. what we have), process indicators (what we do vs. what we should do), and outcome indicators (what we achieved vs. what we should have achieved). Several societies have published lists of indicators, and Table 1 summarizes the main indicators used in each category. Since 2004, the Outcomerea organization has been working on quality indicators for the ICU. A list was built after searching the electronic MEDLINE database using various combinations of the words "adverse event," "iatrogenic," "intensive care unit," "medical error," and "epidemiology." This list contained 180 reported adverse events. In July 2004, we sent the list of 180 events to 30 experts working in 5 ICU fields (cardiovascular disease, neurology, nephrology, pulmonology, and gastroenterology), who added 415 events, for a total of 575 events. Then, 30 other experts including intensivists and ICU nurses participated in a Delphi process to select indicators exhibiting the following characteristics: precise and simple definition of the event and high incidence of the event, impact on morbidity or mortality, and nonpunitive disclosure. A list of 14 events was chosen as sufficiently long to provide useful data yet not so long as to hinder the feasibility of a multicenter study designed to assess their incidence. To reduce bias in data collection, the steering committee developed detailed definitions for all events, and the definitions were then reviewed and validated by the experts. These indicators are listed in Table 1.

**Table 1:** List of safety indicators

Process indicators
Mechanical ventilation
Semi-recumbent position during mechanical ventilation [72,73]
Overinflation of the endotracheal balloon [14]

Sedation
Appropriate sedation [72,74]
Screening for ventilator weaning readiness [73]
Sedation interruption [73]
Sedation monitoring [73]

Medication
Medication administered to wrong patient [14]
Error administering anticoagulant medication [14]
Error prescribing anticoagulant medication [14]
Error administering vasoactive drugs [14]
Error administering insulin [14]
Death or serious disability associated with hypoglycaemia [75]

IV lines
Screening for readiness for removal of central venous catheter [72]

Management
Appropriate use of prophylaxis against gastrointestinal haemorrhage in patients receiving mechanical ventilation [72,73]
Appropriate use of thromboembolism prophylaxis [72,73]
Appropriate use of early enteral nutrition [72]
Early management of severe sepsis, septic shock [72]
Surgical intervention in traumatic brain injury with subdural and/or epidural brain trauma [72]
Monitoring of intracranial pressure in severe traumatic brain injury with abnormal CT findings [72]
Delay in surgical treatment [14]
Change of route for quinolones IV/PO [72]
Screening for MRSA on admission [76]
Pain management in un sedated patients [72]
Events during ICU transport [73]

Complications
Pneumonia associated with mechanical ventilation [72]
Accidental extubation [23,73,76]
Accidental removal of a central venous catheter
Catheter-related bloodstream infections [76] Pneumothorax related to insertion of a central venous catheter [40,76]
Death or serious disability associated with intravascular air embolism [75]
Fall [14]
Death or serious disability associated with a haemolytic reaction due to the administration of ABO-incompatible blood or blood product [75]
Percentage of resistant organisms [74]
Pressure sores [73]

**Outcome indicators**

ICU mortality rate [74]
Hospital mortality rate [73]
Percentage of ICU patients with ICU stays longer than 7 days [74]
Mean ICU length of stay [74]
Mean days on mechanical ventilation [74]
Rate of re-admissions < 72 hours [73]
Family satisfaction [73]

**Structural indicators**

Institutional variables
Process for ensuring staff competencies
Transitional period to integrate new healthcare workers
Clear task identification
Absenteeism, magnitude of personnel turn-over
Adverse-event reporting system
Task variables
Availability of protocols
Policy to prevent medication errors
Policy to register outcomes
Team variables
Adequacy of staffing
Nurse-to-patient ratio
Availability of an intensive care practitioner 24 h a day
Pharmacist present during ICU rounds [77])
Communication or conflicts among team members [78]

The choice of safety indicators depends on several factors, such as previous quality indicators monitored in the unit, monitoring methods, availability of time to monitor additional indicators and to provide feedback to the team, and whether monitoring of processes is instituted before monitoring of outcomes related to those processes. Improving safety requires time, organization, and resources. The goal is to achieve the best possible quality given our resources. Both process and outcome indicators should probably be selected. The process indicators should be related to robust outcomes and the outcomes should be at least partly preventable. Among nosocomial infections, catheter-related infections exhibit these characteristics [21,22]. Other suitable outcomes are accidental extubation [14,23], pressure sores [24,25], falls, rate of readmission within 48 hours [26-28], family satisfaction [29], and morbidity-mortality conferences [30].

### Incidence, risk factors, and impact on patient outcomes of medical errors and adverse events

Comparing the rates of medical errors and adverse events across studies can be challenging due to differences in definitions and to the absence of clear definitions of harms. Even when clear definitions of harms are established before the study, harm rates may be underestimated [14]. Two types of medical errors and adverse events are reported: those related to medications, and those related to procedures or the ICU environment. Administering the right drug to the right patient at the right frequency in the right dose and via the right route represents a challenge for the nursing staff. The Critical Care Safety Study reported an overall rate of 80.5 medication errors associated with harm/1,000 patient-days in medical and coronary-care patients [11]. In the recent worldwide SEE2 study, the rate of parenteral medication errors was 745/1,000 patient-days [10]. With medications given by continuous infusion, the rate was 105/1,000 patient-days [31]. When direct observation at the bedside was used for detection, one medical error was documented for every five doses of medication administered, and among medical errors 23% were errors by omission [17]. Stress ulcer protectors and preventive anticoagulants were among the most often omitted drugs [32]. Vasopressors and catecholamines, insulin, coagulation-altering drugs, antimicrobials, and sedatives were the medications most often involved in medical errors [10]. Insulin and coagulation-altering drugs are associated with numerous errors related to the complexity of dosing and/or monitoring. In recent years, evidence supporting insulin therapy and tight glucose control has led to an increase in the use of insulin in ICU patients [33-35]. Clinical trials have demonstrated that this strategy increases the incidence of hypoglycemic episodes [36-38]. The IATROREF study found a rate of 757 medical errors/1,000 patient-days and 126 adverse events/1,000 patient-days for insulin administration [14].

Numerous other medical errors and adverse events related to procedures and equipment have been investigated in the ICU [9]. Mechanical ventilation was associated with at least one incident in 95/137 patients (0.004 per patient and per day of mechanical ventilation) [39]. Pneumothorax, one of the main complications of both barotrauma and catheter insertion, was reported in 1.5% of patients on day 5 after ventilation initiation [40] and was associated with a threefold increase in the risk of death [15]. All tubes, lines, and drains used in the care of ICU patients can be removed accidentally [41], with an incidence of 22 removals/1,000 patient-days in a French study [42] and 14.5/100 patient-days in a multicenter European study [9]. Maintaining homeostasis is of great importance, and acquired electrolyte disorders can occur as a manifestation of poor quality of care during the ICU stay and can result in increased morbidity or mortality rates [43,44].

Risk factors for medical errors and adverse events have been extensively studied. They pertain either to the ICU or to the patient. The highly sophisticated treatments, technologies, and diagnostic tools used in the ICU are associated with a high risk of medical errors and adverse events [45]. In the IATROREF study, risk factors for medical errors consisted of mechanical ventilation, insulin use, central catheterization, and unscheduled surgery [14]. A study in a French medical ICU identified age older than 65 years and presence of more than two organ failures as independent risk factors for adverse events [13]. A relationship between severity of illness and adverse events was found in a large multicenter European study in which any organ failure, high or excessive workload, and risk factor exposure time independently predicted adverse events [9].

The impact of medical errors or adverse events is difficult to assess due to differences in case-mix, confounding factors for mortality, and occurrence of multiple events in the same patients [15]. Sophisticated analysis methods must therefore be used to evaluate relations between medical errors or adverse events and patient outcomes. The IATROREF study identified 1,192 medical errors in 1,369 patients; of these, 183 (15.4%) in 128 (9.3%) patients were adverse events that were followed by one or more clinical consequences (n = 163) or required one or more procedures or treatments (n = 58). After adjustment for risk-factor exposure time, medical errors, even when multiple, had no impact on mortality. In contrast, having more than two adverse events was associated with a threefold increase in the risk of death [14].

### Preventing medical errors and adverse events

The occurrence of errors is caused by a combination of human factors and system factors [46]. People often make errors, and rates of human error have ranged from 30% to 80% [13,47,48]. What humans do results from interactions between people and the system in which they work. Interventions designed to increase concentration and diligence among healthcare workers are not effective: human errors are unavoidable. Instead, the work conditions should be designed in a way that minimises errors: as stated by Reason, "We cannot change the human condition, but we can change conditions under which humans work" [46]. For example, when two drugs that are very similar in their presentation are stored in the same area, the human-based approach would consist of educating the healthcare workers to pay attention to this similarity to avoid errors. The system-based approach would lead to storage of the two drugs in different places. In the system approach, the key question is not identification of the person responsible for the error but determination of how the error occurred. The mechanism underlying the error is thus identified, without placing blame on the healthcare workers. Then, the organizational flaw can be corrected with the goal of preventing further occurrences of the error.

Since the 1980s, a large amount of work has examined the role for a safety culture in preventing medical errors. Safety culture or safety climate (the two terms are sometimes used interchangeably but "safety culture" is generally seen as a more embracing term than "safety climate") is a concept originally used to describe the safety management inadequacies resulting in major disasters. Thus, the term was first used after the Chernobyl nuclear accident. Now, the concept has evolved to apply to errors at the individual level. The most widely used definition of the safety culture is that developed by the U.K. Health and Safety Commission: the safety culture is "the product of individual and group values, attitudes, perceptions, competencies and patterns of behavior that determine the commitment to, and the style and proficiency of, an organization's health and safety management" [49]. The description of the safety culture concept has been largely empirical. For example, Sexton et al. [50] suggested six dimensions: teamwork climate, job satisfaction, perceptions of management, safety climate, working conditions, and stress recognition; whereas others described a larger number of organizational dimensions [39-41]. ICU and hospital organization is a key point in the safety culture concept. The organizational dimension includes human and technological aspects. Concern over the high rate of medication errors has prompted increased interest in using technology to improve safety [51]. New technologies implemented in recent years include electronic health records, clinical decision support with or without a computerized provider order entry system, bar-code medication administration, and smart infusion pumps [51]. Although these technologies decreased the number of errors, there is little evidence for a concomitant decrease in harms [52-55]. Furthermore, these new technologies have created new errors and harms [56-58]. Before considering their implementation, we must define the clinical settings in which they may be effective, and we must address the specific difficulties raised by their use in the ICU. Many errors are related to less-than-ideal human organization. For instance, burnout syndrome occurs in almost half the physicians and one-third of the nurses in French ICUs [59,60]. Burnout syndrome can adversely affect healthcare worker performance, thereby contributing to medical errors and adverse events. Factors that increase the rate of burnout syndrome include high patient volume, high levels of noise and light, long shifts [61], changes in shift hours, and the occurrence of conflicts [62]. In a study of intern work hours, the traditional intern work schedule involving work shifts longer than 24 hours and a mean of 77 to 81 work hours per week was compared to a schedule designed to decrease sleep deprivation (15-hour shifts at the most, with 60-63 work hours per week) [61]. The traditional schedule was associated with a 22% higher rate of serious errors (193.2 vs. 158.4/1,000 patient-days, *p *< 0.001), a 20.8% higher rate of serious medication errors (99.7 vs. 85.5/1,000 patient-days, *p = *0.03), and a 5.6-fold increase in serious diagnostic errors (18.6 vs. 3.3/1,000 patient-days, *p *< 0.001). It would be useful to test interventions designed to improve well-being at work and to assess their impact on the rates of medical errors and adverse events [63].

A number of targets for improvement have been identified [64,65]. The Agency for Healthcare Research and Quality identified five measures that have effects of varying magnitude on physician behavior (academic detailing, audit and feedback, reminder systems, interventions by local opinion leaders, and printed material) [65]. Multifaceted programs or bundles are more effective to improve safety than are isolated measures and have been used in several studies [19,66,67]. In the IATROREF study, we used educational slide shows, printed educational material, and feedback meetings; and we focused on errors administering insulin, errors administering and prescribing anticoagulants, and accidental removal of endotracheal tubes and central venous catheters [19]. Our program was effective in preventing insulin errors and accidental tube/catheter removal. However, significant Hawthorne effects were documented [19]. In a multicenter, cluster-randomized study, a multifaceted program, including feedback meetings, expert-led educational sessions, and dissemination of algorithms, significantly improved process indicators, such as the semirecumbent position for nosocomial pneumonia prevention and measures to prevent central catheter infections [67]. Similarly, a bundle strategy decreased the rate of nosocomial pneumonia [68]. In 2003, the Michigan Keystone ICU Patient Safety Program based on a John Hopkins University model was launched to eliminate catheter-related infections and ventilator-associated pneumonia. The model is based on the four Es: Engage, Educate, Execute, and Evaluate [69]. The Michigan hospitals reported improvements in adherence to guidelines for ventilator-associated pneumonia prevention and decreases in the rates of catheter-related infections [22,70,71].

## Conclusions

Medical errors and adverse events are very common in ICUs, and among them the most prevalent involve medications. Identification of these errors requires efficient reporting systems, usually based on a combination of methods. Many valid indicators have been developed. The prevention of medical errors and adverse events requires combined changes in ICU organization and healthcare worker behaviors. Sharing values and behaviors within the team with the support of hospital leaders is probably the most powerful means of building a safety climate for the patients. Multilayered programs associated with a profound change in the approach to patient safety offer the greatest likelihood of success.

## Competing interests

The authors declare that they have no competing interests.

## Authors' contributions

MG drafted the manuscript. FP, CB, AM, NL, and BM critically revised the manuscript for important intellectual content and approved the final version of the manuscript submitted for publication. All authors read and approved the final manuscript.
